# Impact of glycemic control on the progression of aortic stenosis: a single-center cohort study using a common data model

**DOI:** 10.1186/s12902-023-01403-5

**Published:** 2023-07-10

**Authors:** In-Chang Hwang, Seok Kim, Dachung Boo, Changhyun Park, Sooyoung Yoo, Yeonyee E. Yoon, Goo-Yeong Cho

**Affiliations:** 1 Department of Cardiology, Cardiovascular Center, Seoul National University Bundang Hospital, 82 Gumi-Ro-173-Gil, Seongnam, Gyeonggi 13620 South Korea; 2 Department of Internal Medicine, Seoul National University College of Medicine, Seoul, Korea; 3 Office of eHealth Research and Business, Seoul National University Bundang Hospital, Seongnam, Gyeonggi Korea

**Keywords:** Aortic stenosis, Diabetes mellitus, Glycemic control

## Abstract

**Background:**

Diabetes mellitus (DM) is a well-established risk factor for the progression of degenerative aortic stenosis (AS). However, no study has investigated the impact of glycemic control on the rate of AS progression. We aimed to assess the association between the degree of glycemic control and the AS progression, using an electronic health record-based common data model (CDM).

**Methods:**

We identified patients with mild AS (aortic valve [AV] maximal velocity [Vpeak] 2.0–3.0 m/sec) or moderate AS (Vpeak 3.0–4.0 m/sec) at baseline, and follow-up echocardiography performed at an interval of ≥ 6 months, using the CDM of a tertiary hospital database. Patients were divided into 3 groups: no DM (*n* = 1,027), well-controlled DM (mean glycated hemoglobin [HbA1c] < 7.0% during the study period; *n* = 193), and poorly controlled DM (mean HbA1c ≥ 7.0% during the study period; *n* = 144). The primary outcome was the AS progression rate, calculated as the annualized change in the Vpeak (△Vpeak/year).

**Results:**

Among the total study population (*n* = 1,364), the median age was 74 (IQR 65–80) years, 47% were male, the median HbA1c was 6.1% (IQR 5.6–6.9), and the median Vpeak was 2.5 m/sec (IQR 2.2–2.9). During follow-up (median 18.4 months), 16.1% of the 1,031 patients with mild AS at baseline progressed to moderate AS, and 1.8% progressed to severe AS. Among the 333 patients with moderate AS, 36.3% progressed to severe AS. The mean HbA1c level during follow-up showed a positive relationship with the AS progression rate (β = 2.620; 95% confidence interval [CI] 0.732–4.507; *p* = 0.007); a 1%-unit increase in HbA1c was associated with a 27% higher risk of accelerated AS progression defined as △Vpeak/year values > 0.2 m/sec/year (adjusted OR = 1.267 per 1%-unit increase in HbA1c; 95% CI 1.106–1.453; *p* < 0.001), and HbA1c ≥ 7.0% was significantly associated with an accelerated AS progression (adjusted odds ratio = 1.524; 95% CI 1.010–2.285; *p* = 0.043). This association between the degree of glycemic control and AS progression rate was observed regardless of the baseline AS severity.

**Conclusion:**

In patients with mild to moderate AS, the presence of DM, as well as the degree of glycemic control, is significantly associated with accelerated AS progression.

**Supplementary Information:**

The online version contains supplementary material available at 10.1186/s12902-023-01403-5.

## Background

Degenerative aortic stenosis (AS) is a progressive disease, in which the main pathophysiology is atherosclerosis with inflammation, fibrosis, and calcification of the aortic valve (AV) [1, 2]. The presence of diabetes mellitus (DM) contributes to the development and progression of AS, through the augmentation of proinflammatory processes, enhanced lipid accumulation, and accelerated calcification of valvular endothelial and interstitial cells [3]. These pathophysiologic links have been confirmed in clinical studies, which showed a significant association between the presence of DM and the risk of developing AS, as well as the progression of AS [4–7].

Although the association between the presence of DM and accelerated AS progression is well-established, (3-7) no study has investigated the association between the degree of glycemic control and the rate of AS progression. Based on the underlying pathophysiology of AS progression, it could be expected that patients with poorly-controlled DM have more rapid AS progression. In a recent study of AV specimens from patients undergoing AV replacement, increased expression of valvular advanced glycation end products (AGEs) was associated with AS severity, suggesting that the level of glycemic control might be associated with AS progression rate [8]. This association would have clinical significance, given the lack of effective medical therapy to prevent AS progression [9].

In the present study, we aimed to assess the association between the degree of glycemic control and AS progression rate in patients with mild (defined as an AV maximal velocity [Vpeak] of 2.0–3.0 m/sec) or moderate AS (defined as a Vpeak of 3.0–4.0 m/sec), using an electronic health record (EHR)-based common data model (CDM).

## Methods

### Data sources

This study utilized Observational Health Data Sciences and Informatics (OHDSI) open source software and the Observational Medical Outcomes Partnership (OMOP)-CDM version 5.3. The CDM comprised de-identified patient-level EHR data from outpatients and inpatients was obtained between April 2003 and July 2019, routinely collected during medical services at Seoul National University Bundang Hospital. The study protocol was approved by our institutional review board (B-1812–510-111) on December 26, 2018, which waived the requirement of informed consent. All aspects of the study were conducted in accordance with the Declaration of Helsinki.

### Study population

**Fig. 1:**
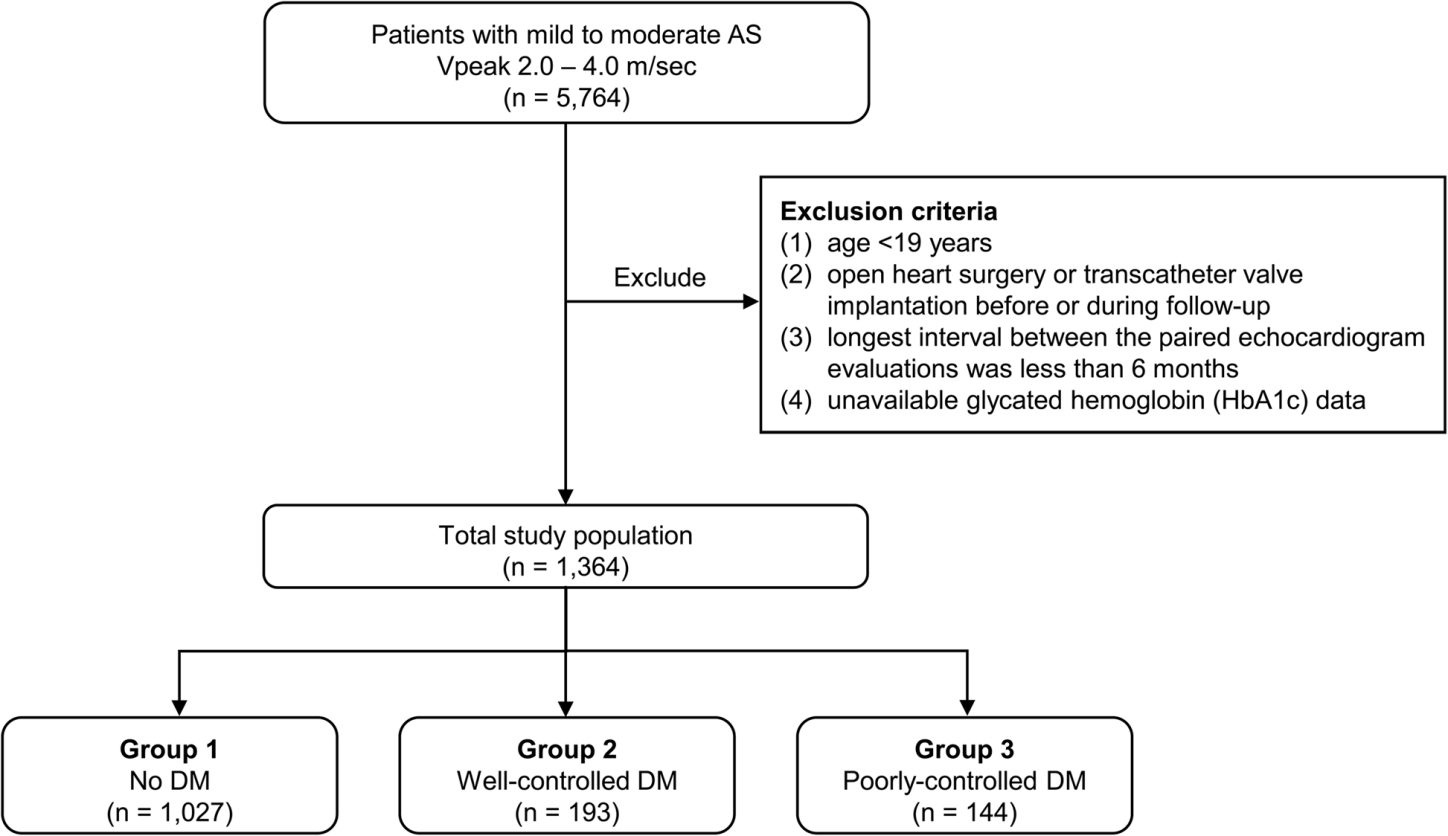
Study attrition diagram. Abbreviations: AS, aortic stenosis; DM, diabetes mellitus

From the CDM database, we extracted patients with mild (defined as a Vpeak/] of 2.0–3.0 m/sec) or moderate AS (defined as a Vpeak of 3.0–4.0 m/sec) at baseline, and for whom follow-up echocardiography was performed at an interval of ≥ 6 months (*n* = 5,764) (Fig. 1). Considering the intermittent changes in the antidiabetic medications and the fluctuations of HbA1c levels, we selected the first 2 echocardiograms (baseline showing mild to moderate AS, and follow-up at an interval of ≥ 6 months), even in patients who underwent three or more echocardiographic examinations.

The exclusion criteria were as follows: (1) age < 19 years; (2) open heart surgery or transcatheter valve implantation before or during follow-up; (3) longest interval between the paired echocardiogram evaluations was less than 6 months; and (4) unavailable glycated hemoglobin (HbA1c) data (Fig. 1). After applying these exclusion criteria, a total of 1,364 patients were included in the final analysis.

### Definitions of covariates

In the present study, the severity of AS was defined using the Vpeak, for which the Logical Observation Identifiers Names and Codes (LOINC) was used to represent the echocardiographic finding in the CDM (Supplementary Table S1). For the exclusion of patients who underwent aortic valve replacement, the Systematized Nomenclature of Medicine Clinical Terms (SNOMED-CT) were used to define the procedure. Other echocardiographic parameters, including left ventricular (LV) end-diastolic and end-systolic dimensions, LV end-diastolic and end-systolic volumes, LV mass index, the mean pressure gradient across the AV (meanPG), and the AV area (AVA) were also mapped to the LOINC concepts in the CDM (Supplementary Table S1).

The overall attrition diagram and a detailed list of concept sets and International Classification of Disease (ICD) codes used for constructing target definitions and exclusion criteria are provided in Supplementary Table S1. Patients with DM were designated by the SNOMED-CT, current use of antidiabetic medication, and a fasting glucose ≥ 126 mg/dL or HbA1c ≥ 6.5%. “Well-controlled DM” was defined as a mean HbA1c level < 7.0% during the study period, and “poorly-controlled DM” was defined as a mean HbA1c level ≥ 7.0% during the study period. Patients meeting the above eligibility criteria and definitions were identified using ATLAS version 2.8.0 and Structured Query Language (SQL) codes; outliers, as determined by R programming version 4.0.3 (The R Foundation for Statistical Computing, Vienna, Austria), were excluded. The presence of hypertension, dyslipidemia, heart failure, and atrial fibrillation was defined using the SMONED-CT for CDM condition data, and the use of medication was defined using RxNorm, RxNorm Extension, and Anatomical Therapeutic Chemical Classification System (ATC) for CDM drug exposure data.

### Echocardiography

All echocardiographic images were obtained using a standard ultrasound machine with a 2.5-MHz probe. Standard techniques were used to obtain M-mode, 2-dimensional, and Doppler measurements, in accordance with the American Society of Echocardiography’s guidelines [10, 11]. The Vpeak was recorded using the apical, right parasternal, or suprasternal window yielding the highest velocity signal. The AV meanPG was calculated using a modified Bernoulli equation, and the AVA was estimated from the continuity equation using the left ventricular outflow tract diameter and flow velocity.

### Study outcomes

The primary outcome was the AS progression rate, calculated as the annualized change in the Vpeak (△Vpeak/year). Annualized changes in the meanPG (△meanPG/year) and AVA (△AVA/year) were also considered; however, due to missing AVA values in the dataset, the change in AVA was not assessed as a study outcome.

In order to evaluate the impact of the presence of DM and the degree of glycemic control on the AS progression rate, the study population was divided into three groups: patients without DM (no DM; *n* = 1,027), patients with well-controlled DM (HbA1c < 7.0%; *n* = 193), and patients with poorly-controlled DM (HbA1c ≥ 7.0%: *n* = 144).

### Statistical analysis

Continuous variables are presented as the median with interquartile range (IQR) after testing for normality with Shapiro–Wilk test, and categorical variables as frequencies. Differences between groups were evaluated using the Kruskal–Wallis test or one-way analysis of variance (ANOVA) with Bonferroni correction for multiple comparisons for continuous variables, and the χ^2^ test for categorical variables. Bonferroni correction was performed to compare between the three groups as a post hoc test if the ANOVA test result was significant, in order to avoid bias of multiple testing.

Trends in the AS progression rate (△Vpeak/year) according to the presence of DM and degree of glycemic control, as well as the baseline Vpeak, were assessed using the penalized smoothing spline methods. The association between the degree of glycemic control and AS progression rate (△Vpeak/year) was assessed using linear regression modeling, using the △Vpeak/year as a dependent variable. Because the value of the dependent variable was numerically small, the dependent variable (△Vpeak/year) was analyzed in units of ‘cm/sec/year’. Demographic factors, laboratory findings including the mean HbA1c level during follow-up, and echocardiographic findings were used as independent variables. Because of the number of variables used in the univariable linear regression analysis was very large compared to the number of stud population, and there is possible multicollinearity between the variables, variables with a *P*-value of < 0.1 on univariable analysis were entered into the multivariable regression analysis, using the stepwise backward elimination with the Akaike Information Criterion (AIC) method [12, 13]. Additionally, as the highest △Vpeak/year tertile among patients with DM in the present study was 0.2 m/sec/year, patients with △Vpeak/year values > 0.2 m/sec/year were considered to indicate accelerated AS progression. The association between the degree of glycemic control and accelerated AS progression (the dependent variable; defined as △Vpeak/year values > 0.2 m/sec/year) was analyzed using a single-level logistic regression modeling, using demographic factors, laboratory findings and echocardiographic findings as independent variables. Variables with a *P*-value of < 0.1 on univariable analysis were entered into the multivariable regression analysis, using the stepwise backward elimination with the AIC method [12, 13].

Data were analyzed using R programming version 4.0.3 (The R Foundation for Statistical Computing, Vienna, Austria). A two-sided *p*-value of < 0.05 was considered statistically significant.

## Results

### Baseline characteristics

Baseline characteristics, laboratory findings, and echocardiography data of the total study population are summarized in Table 1. The median age was 74 (IQR 65–80) years and 47% were male. Compared to those without DM (group 1), the patients with DM had higher frequencies of comorbidities, which were more frequent in those with poorly-controlled DM (group 3) than in those with well-controlled DM (group 2).

**Table 1:** Baseline characteristics and the rates of AS progression

	**Total study population** **(*n* = 1,364)**	**Group 1** **No DM** **(*n* = 1,027)**	**Group 2** **DM, well-controlled** **(*n* = 193)**	**Group 3** **DM, poorly-controlled** **(*n* = 144)**	**Overall *P***
Age (years)	74 (65 – 80)	73 (63 – 79)	76 (70 – 81)	75 (70 – 80)	< 0.001
Male sex	645 (47.3%)	470 (45.8%)	108 (56.0%)	67 (46.5%)	0.033
Body-mass index (kg/m^2^)	24.2 (22.2 – 26.7)	24.0 (22.1 – 26.3)	24.5 (22.3 – 27.5)	24.9 (23.0 – 27.9)	< 0.001
Body-surface area (m^2^)	1.6 (1.5 – 1.8)	1.6 (1.5 – 1.8)	1.7 (1.6 – 1.8)	1.7 (1.6 – 1.8)	0.002
Systolic blood pressure (mmHg)	129 (116 – 142)	128 (116 – 141)	129 (118 – 146)	134 (118 – 149)	0.015
Diastolic blood pressure (mmHg)	70 (63 – 79)	71 (64 – 79)	68 (62 – 75)	68 (60 – 77)	0.006
Heart rate (bpm)	72 (64 – 84)	72 (64 – 85)	73 (64 – 83)	72 (65 – 82)	0.798
Duration of diabetes (years)	N/A	N/A	4.3 (0.8 – 7.5)	4.9 (0.3 – 8.6)	N/A
Hypertension	379 (27.8%)	247 (17.4%)	85 (6.0%)	47 (3.3%)	< 0.001
Dyslipidemia	177 (13.0%)	99 (7.0%)	43 (3.0%)	35 (2.5%)	< 0.001
Coronary artery disease	269 (19.7%)	177 (12.5%)	52 (3.7%)	40 (2.8%)	< 0.001
Heart failure	124 (9.1%)	82 (5.8%)	25 (1.8%)	17 (1.2%)	0.043
Atrial fibrillation	153 (11.2%)	109 (7.7%)	25 (1.8%)	19 (1.3%)	0.466
**Laboratory findings**					
Hemoglobin (g/dL)	12.4 (10.8 – 13.7)	12.6 (11.2 – 13.9)	11.4 (10.3 – 13.2)	11.5 (10.1 – 13.1)	< 0.001
Creatinine (mg/dL)	0.9 (0.7 – 1.2)	0.9 (0.7 – 1.1)	1.1 (0.8 – 1.6)	1.0 (0.8 – 1.5)	< 0.001
GFR (mL/min/1.73m^2^)	75.9 (51.7 – 95.9)	82.5 (58.8 – 97.1)	60.2 (35.8 – 85.0)	64.8 (36.3 – 88.0)	< 0.001
Total cholesterol (mg/dL)	158 (133 – 187)	164 (139 – 193)	143 (122 – 168.5)	141 (127 – 164)	< 0.001
Triglyceride (mg/dL)	103 (77 – 144)	99 (74 – 142)	112.5 (76 – 146.5)	113 (84.8 – 173)	0.007
HDL cholesterol (mg/dL)	47 (38 – 57)	49 (41 – 60)	42 (36 – 53)	41 (35 – 49)	< 0.001
LDL cholesterol (mg/dL)	89 (71 – 109)	95 (75 – 116)	83 (65 – 100)	79 (63 – 97)	< 0.001
HbA1c (%) at baseline	6.1 (5.6 – 6.9)	5.8 (5.4 – 6.1)	6.4 (5.9 – 6.8)	7.6 (7.0 – 8.6)	< 0.001
Prior HbA1c (%)^a^	6.4 (5.7 – 6.9)	5.9 (5.5 – 6.1)	6.7 (6.2 – 7.0)	7.8 (7.1 – 8.3)	< 0.001
Fasting glucose (mg/dL)	105 (93 – 129)	101 (92 – 114)	118 (100 – 144)	141 (113.5 – 173)	< 0.001
Variability of fasting glucose during follow-up					
Standard deviation of fasting glucose (SD_FG_)	13.2 (6.1 – 26.6)	8.5 (4.7 – 18.8)	15.5 (8.5 – 28.3)	30.7 (17.1 – 52.1)	< 0.001
Coefficient of variation of fasting glucose (CV_FG_)	11.5 (6.1 – 22.0)	8.3 (4.9 – 16.3)	13.3 (7.7 – 22.3)	22.9 (13.0 – 34.1)	< 0.001
Average real variation of fasting glucose (ARV_FG_)	0.3 (-4.5 – 4.0)	0.3 (-3.0 – 3.8)	0.3 (-5.3 – 3.2)	0.4 (-12.8 – 8.9)	0.770
**Echocardiographic parameters**					
LVEF (%)	62.6 (57.1 – 67.2)	62.7 (57.4 – 67.2)	62.3 (55.7 – 67.2)	62.3 (55.7 – 67.2)	0.381
LVMI (g/m2)	108.4 (91.4 – 130.2)	108.1 (90.7 – 130.6)	113.6 (94.5 – 133.1)	106.6 (95.5 – 125.3)	0.311
LAVI (mL/m2)	42.8 (32.6 – 60.0)	42.7 (32.3 – 60.5)	44.1 (34.4 – 60.8)	44 (32.3 – 57.8)	0.981
Vpeak (m/sec)	2.5 (2.2 – 2.9)	2.5 (2.2 – 3.0)	2.4 (2.2 – 2.8)	2.4 (2.2 – 2.9)	0.017
meanPG (mmHg)	13.0 (10.1 – 19.4)	13.3 (10.4 – 20.0)	12.5 (10.0 – 17.1)	12.9 (10.0 – 18.5)	0.075
AVA (cm^2^)	1.3 (1.1 – 1.6)	1.3 (1.1 – 1.6)	1.3 (1.2 – 1.6)	1.3 (1.1 – 1.4)	0.293
**Medication**					
RAS blocker	462 (33.9%)	309 (30.1%)	96 (49.7%)	57 (39.6%)	< 0.001
Beta blocker	294 (21.6%)	194 (18.9%)	56 (29.0%)	44 (30.6%)	< 0.001
Calcium channel blocker	325 (23.8%)	203 (19.8%)	76 (39.4%)	46 (31.9%)	< 0.001
Spironolactone	70 (5.1%)	53 (5.2%)	10 (5.2%)	7 (4.9%)	0.988
Statins	458 (33.6%)	291 (28.3%)	84 (43.5%)	83 (57.6%)	< 0.001
Warfarin	139 (10.2%)	116 (11.3%)	16 (8.3%)	7 (4.9%)	0.037
DOAC	37 (2.7%)	24 (2.3%)	8 (4.2%)	5 (3.5%)	0.307
Antiplatelet agents	448 (32.8%)	284 (27.7%)	89 (46.1%)	75 (52.1%)	< 0.001
Metformin	105 (7.7%)	0 (0.0%)	60 (31.2%)	45 (31.3%)	< 0.001
DPP4 inhibitors	65 (4.8%)	0 (0.0%)	31 (16.1%)	34 (23.6%)	< 0.001
SGLT2 inhibitors	1 (0.1%)	0 (0.0%)	1 (0.5%)	0 (0.0%)	0.048
Sulfonylurea	78 (5.7%)	0 (0.0%)	41 (21.2%)	37 (25.7%)	< 0.001
Thiazolidinedione	6 (0.4%)	0 (0.0%)	2 (1.0%)	4 (2.8%)	< 0.001
Alpha glucosidase inhibitor	12 (0.9%)	0 (0.0%)	7 (3.6%)	5 (3.5%)	< 0.001
AS severity at baseline					
Mild AS	1031 (75.6%)	766 (74.6%)	154 (79.8%)	111 (77.1%)	0.275
Moderate AS	333 (24.4%)	261 (25.4%)	39 (20.2%)	33 (22.9%)	0.807
AS severity at baseline					
Mild AS	878 (64.4%)	653 (63.6%)	134 (69.4%)	91 (63.2%)	0.211
Moderate AS	346 (25.4%)	261 (25.4%)	42 (21.8%)	43 (29.9%)	0.239
Severe AS	140 (10.3%)	113 (11.0%)	17 (8.8%)	10 (6.9%)	0.250
**AS progression rate**					
Follow-up interval (months)	18.4 (12.3 – 31.4)	18.6 (12.4 – 31.8)	18.6 (12.3 – 27.8)	16.7 (11.6 – 29.0)	0.199
△Vpeak/year (m/sec/year)	0.064 (-0.034 – 0.225)	0.057 (-0.042 – 0.225)	0.071 (0.000 – 0.205)	0.092 (-0.002 – 0.273)	0.015
△meanPG/year (mmHg/year)	0.705 (-0.493 – 2.931)	0.585 (-0.624 – 2.812)	0.991 (-0.229 – 2.638)	1.178 (-0.156 – 3.259)	0.059

Values are given as the mean with standard deviation or as a number (percentage)

*Abbreviations*: *AS* aortic stenosis, *DM* diabetes mellitus, *GFR* glomerular filtration rate, *HbA1c* glycated hemoglobin, *HDL* high-density lipoprotein, *LDL* low-density lipoprotein, *LVEF* left ventricular ejection fraction, *LVMI* left ventricular mass index, *LAVI* left atrial volume index, *Vpeak* aortic valve maximal velocity, *meanPG* mean pressure gradients across the aortic valve, *AVA* aortic valve area, *RAS* renin-angiotensin system, *DOAC* direct oral anticoagulants, *DPP4* dipeptidyl peptidase-4, *SGLT2* sodium-glucose cotransporter 2, *N/A* not applicable

^a^Prior HbA1c (%) indicates the median HbA1c levels measured before 3 months and 1 year prior to the inclusion

The median HbA1c levels were 5.8% (IQR 5.4–6.1) in patients without DM, 6.4% (IQR 5.9–6.8) in patients with well-controlled DM, and 7.6% (IQR 7.0–8.6) in patients with poorly-controlled DM. At baseline, the median Vpeak, meanPG, and AVA were 2.5 m/sec (IQR 2.2–2.9), 13.1 mmHg (IQR 10.2–19.4), and 1.3 cm^2^ (IQR 1.1–1.6) in total study population, and the severity of AS was similar between the 3 groups.

### Association between glycemic control and AS progression

The rate of AS progression was different between the groups: the △Vpeak/year was 0.06 m/sec/year (IQR -0.04–0.23) in patients without DM, 0.07 m/sec/year (IQR 0.00–0.21) in patients with well-controlled DM, and 0.09 m/sec/year (IQR 0.00–0.27) in those with poorly-controlled DM (overall *p* = 0.015) (Table 1). The rate of AS progression assessed by the changes in the meanPG and AVA showed similar trends of a lower AS progression rate in patients without DM, and a higher AS progression rate in patients with DM, especially in those with poorly-controlled DM.

**Fig. 2:**
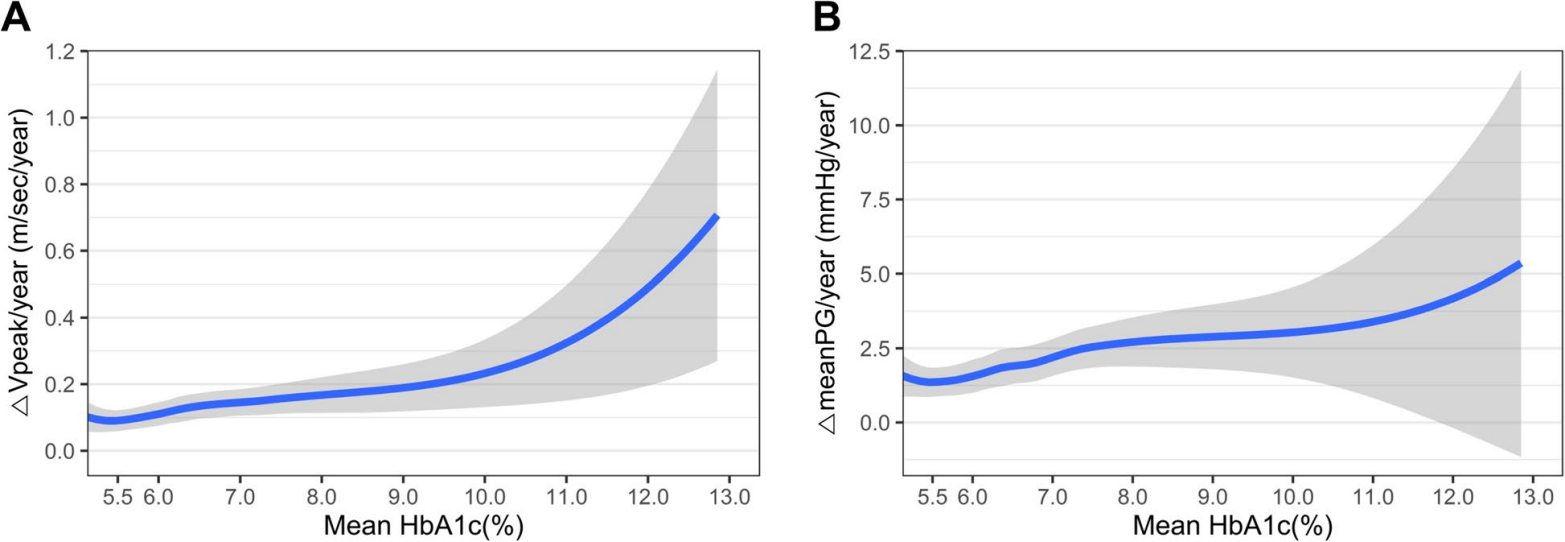
Trends in AS progression according to the degree of glycemic control. The association between the mean HbA1c levels and the AS progression rate, as assessed by the change in (**A**) Vpeak and (**B**) meanPG, is shown. Abbreviations: AS, aortic stenosis; Vpeak, aortic valve maximal velocity; meanPG, mean pressure gradients across the aortic valve

In order to evaluate the overall impact of glycemic control on the rate of AS progression, the △Vpeak/year and △meanPG/year were plotted according to the mean HbA1c levels in Fig. 2. The AS progression rate was proportional to the degree of glycemic control, showing a more rapid progression in those with a higher HbA1c level. The association between the degree of glycemic control and the rate of AS progression (△Vpeak/year; cm/sec/year) was further assessed in linear regression analyses (Table 2 and Supplementary Table S2). In the multivariable regression model, the higher mean HbA1c level during follow-up (*β* = 2.620; 95% CI 0.732–4.507; *p* = 0.007), higher Vpeak at baseline (*β* = 5.574; 95% CI 1.329–9.818; *p* = 0.010), higher total cholesterol level at baseline (*β* = 0.063; 95% CI 0.005–0.120; *p* = 0.034), and the presence of coronary artery disease (*β* = 6.716; 95% CI 1.344–12.088; *p* = 0.014) were significantly associated with the higher rate of AS progression; whereas the presence of heart failure was negatively associated with the rate of AS progression (Table 2).

**Table 2:** Multivariable linear regression models for the predictors of AS progression rate

	**β**	**95% CI**	***P* value**
CAD	6.716	1.344 – 12.088	0.014
Heart failure	-9.952	-17.725 – -2.178	0.012
Total cholesterol (per + 1 mg/dL)	0.063	0.005 – 0.120	0.034
Mean HbA1 during follow-up (per + 1%-unit increase in HbA1c)	2.620	0.732 – 4.507	0.007
Vpeak (per + 1 m/sec)	5.574	1.329 – 9.818	0.010

Because the value of the dependent variable was numerically small, the dependent variable (△Vpeak/year) was analyzed in units of ‘cm/sec/year’

Univariable factors with *P*-values < 0.1 were entered into the multivariable linear regression analysis, using the stepwise backward elimination with the Akaike Information Criterion (AIC) method. Variables with significant association with AS progression (△Vpeak/year) are shown

*Abbreviations*: *CI* confidence interval, *CAD* coronary artery disease, *HbA1c* glycated hemoglobin, *Vpeak* aortic valve maximal velocity

**Fig. 3:**
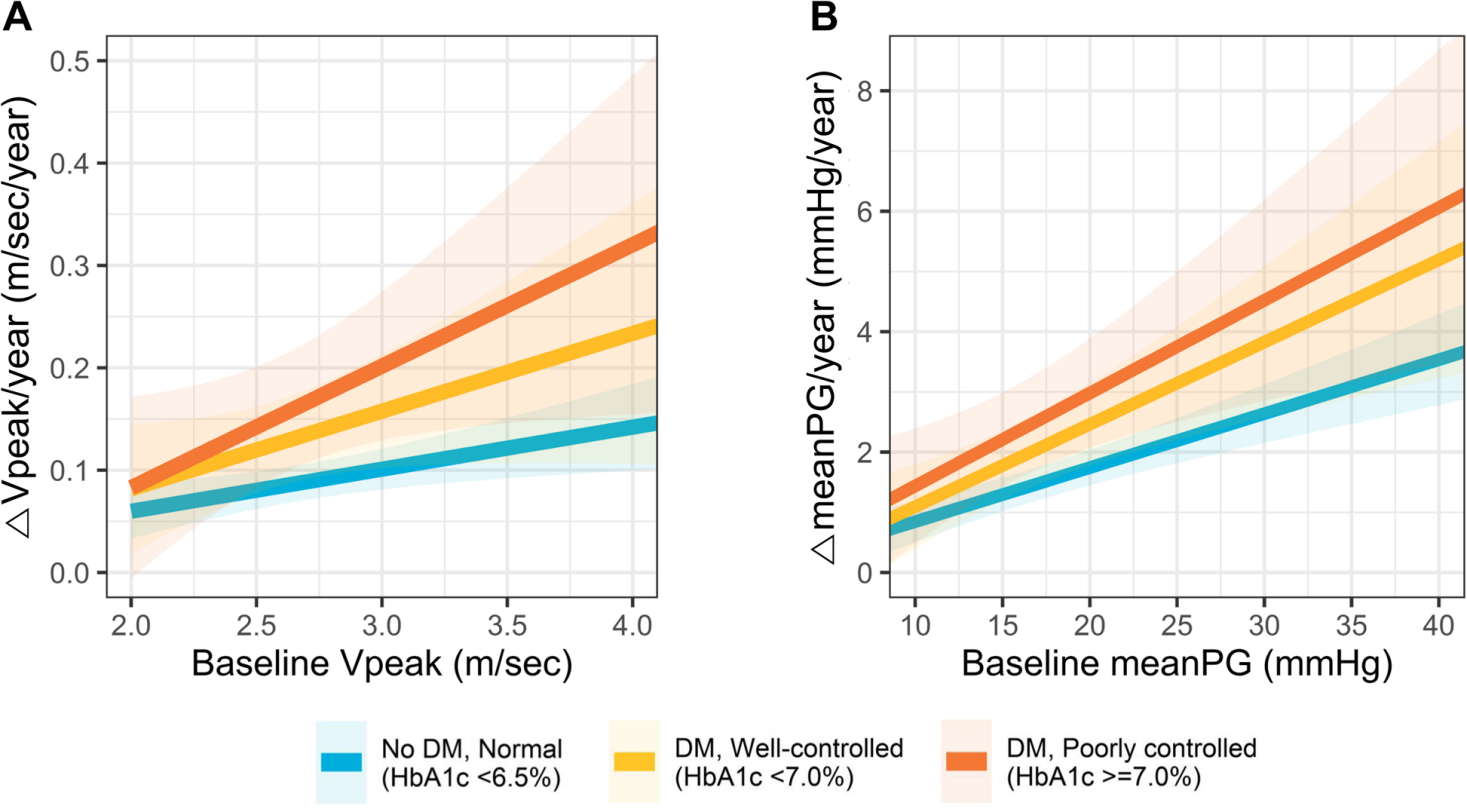
Differences in AS progression according to the presence of diabetes mellitus and degree of glycemic control. The AS progression rates, as assessed by the change in (**A**) Vpeak and (**B**) meanPG, is shown across the range of baseline AS severity in patients without DM, with well-controlled DM, and with poorly-controlled DM

Considering that the baseline Vpeak was significantly associated with the rate of AS progression, we compared the rate of AS progression between the patients without DM, those with well-controlled DM, and those with poorly-controlled DM, across the range of baseline Vpeak (Fig. 3). As the baseline Vpeak increases, the rate of AS progression rate showed a tendency to gradually increase (Fig. 3A). Of note, compared to those without DM, the increase in the AS progression rate according to the baseline Vpeak value showed a steeper pattern in patients with DM, and the slope was the steepest in those with poorly-controlled DM. The same trend was observed, when the AS progression rate was assessed using the changes in the meanPG (Fig. 3B).

Using the △Vpeak/year value of > 0.2 m/sec/year as the cutoff for accelerated AS progression, we performed logistic regression analyses to investigate the association between the glycemic control and the accelerated AS progression (Table 3 and Supplementary Table S3). In the multivariable logistic regression analysis, a + 1%-unit increase in the mean HbA1c level was associated with a 27% higher risk of accelerated AS progression (adjusted OR = 1.267 per + 1%-unit increase in HbA1c; 95% CI 1.106–1.453; *p* < 0.001). This association maintained its statistical significance when the glycemic control level was used as a categorical variable (mean HBA1c ≥ 7.0%) (adjusted OR = 1.524; 95% CI 1.010–2.285; *p* = 0.043).

**Table 3:** Multivariable logistic regression models for the predictors of accelerated AS progression

	**Assessed as continuous variables**			**Assessed as categorical variables**		
	**Adjusted OR**	**95% CI**	***P* value**	**Adjusted OR**	**95% CI**	***P* value**
Male sex	1.539	1.092 – 2.177	0.014	-	-	-
Total cholesterol (per + 1 mg/dL)	1.007	1.002 – 1.011	0.002	1.006	1.002 – 1.010	0.005
Mean HbA1 during follow-up (per + 1%-unit increase in HbA1c)	1.267	1.106 – 1.453	< 0.001	-	-	-
HbA1c ≥ 7.0%	-	-	-	1.524	1.010 – 2.285	0.043
Vpeak (per + 1 m/sec)	1.659	1.233 – 2.231	< 0.001	-	-	-
Vpeak ≥ 3 m/sec	-	-	-	1.696	1.151 – 2.487	0.007
Use of RAS blocker	0.657	0.454 – 0.943	0.024	0.654	0.453 – 0.935	0.021
Use of spironolactone	-	-	-	0.293	0.068 – 0.865	0.050

Univariable factors with *P*-values < 0.1 were entered into the multivariable logistic regression analysis, using the stepwise backward elimination with the AIC method. Variables with significant association with accelerated AS progression (△Vpeak/year > 0.2 m/sec/year) are shown

*Abbreviations*: *OR* odds ratio, *CI*, confidence interval, *HbA1c* glycated hemoglobin, *Vpeak* aortic valve maximal velocity, *RAS* renin-angiotensin system

## Discussion

In the present study, we assessed the AS progression rate according to the presence of DM and degree of glycemic control among 1,364 patients with mild to moderate AS, using an EHR-based CDM model. The presence of DM, as well as the glycemic control level, was significantly associated with accelerated AS progression. To our knowledge, this is the first study to demonstrate an association between the degree of glycemic control and the AS progression rates. Our findings highlight the importance of glycemic control in patients with DM and mild to moderate AS.

The presence of DM is a well-established risk factor for AS development and progression. A study of 6,780 participants from the Multi-Ethnic Study of Atherosclerosis (MESA) showed that patients with DM have a 1.7–2.1 fold higher risk of AV calcification, as detected on computed tomography [4]. Furthermore, *Kamalesh *et al. evaluated the change in AVA in 166 patients with AS, and found that among those with moderate AS at baseline, patients with DM had a larger reduction in AVA than patients without DM [5]. More recently, large-scale population studies have confirmed the significant impact of DM on AS progression. For example, the Cardiovascular Health in Ambulatory Care Research Team (CANHEART) study assessed the risk of incident severe AS in 1.12 million individuals during a median of 13 years, and reported that the presence of DM increased the risk of developing severe AS by 50% [7]. Similarly, a study by *Larsson *et al. assessed the association of DM with seven cardiovascular diseases among more than 70,000 adults, and demonstrated that the presence of type 2 DM increased the risk of incident AS by 34% [6].

The robust association between the presence of DM and accelerated AS progression can be explained by various pathophysiologic mechanisms. Patients with DM have a higher expression of proinflammatory C-reactive protein (CRP) in AV tissue, and higher levels of CRP and tissue factor in plasma, than patients without DM, suggesting that increased proinflammatory processes lead to accelerated AS progression [14, 15]. In addition, transient hyperglycemia has been shown to lead to excessive proinflammatory phospholipid synthesis and coagulation activation in valvular interstitial cells, supporting the impact of proinflammatory signals on accelerated AS progression in patients with DM [16].

Although the impact of the presence of DM on AS progression has been well-established, the impact of the degree of glycemic control on AS progression was largely unknown prior to the present study. In a recent study by *Kopytek *et al., patients with DM showed increased expression of AGEs and AGE receptors in AV tissue, which was correlated with the HbA1c level [8]. Although this finding suggests that patients with poorly-controlled DM have accelerated AS progression, the AS progression rate was not assessed in this previous study. Considering the beneficial effects of strict glycemic control on the prevention of major cardiovascular adverse events, and the underlying pathophysiology, we thought it reasonable to hypothesize that the degree of glycemic control is associated with the AS progression rate.

**Fig. 4:**
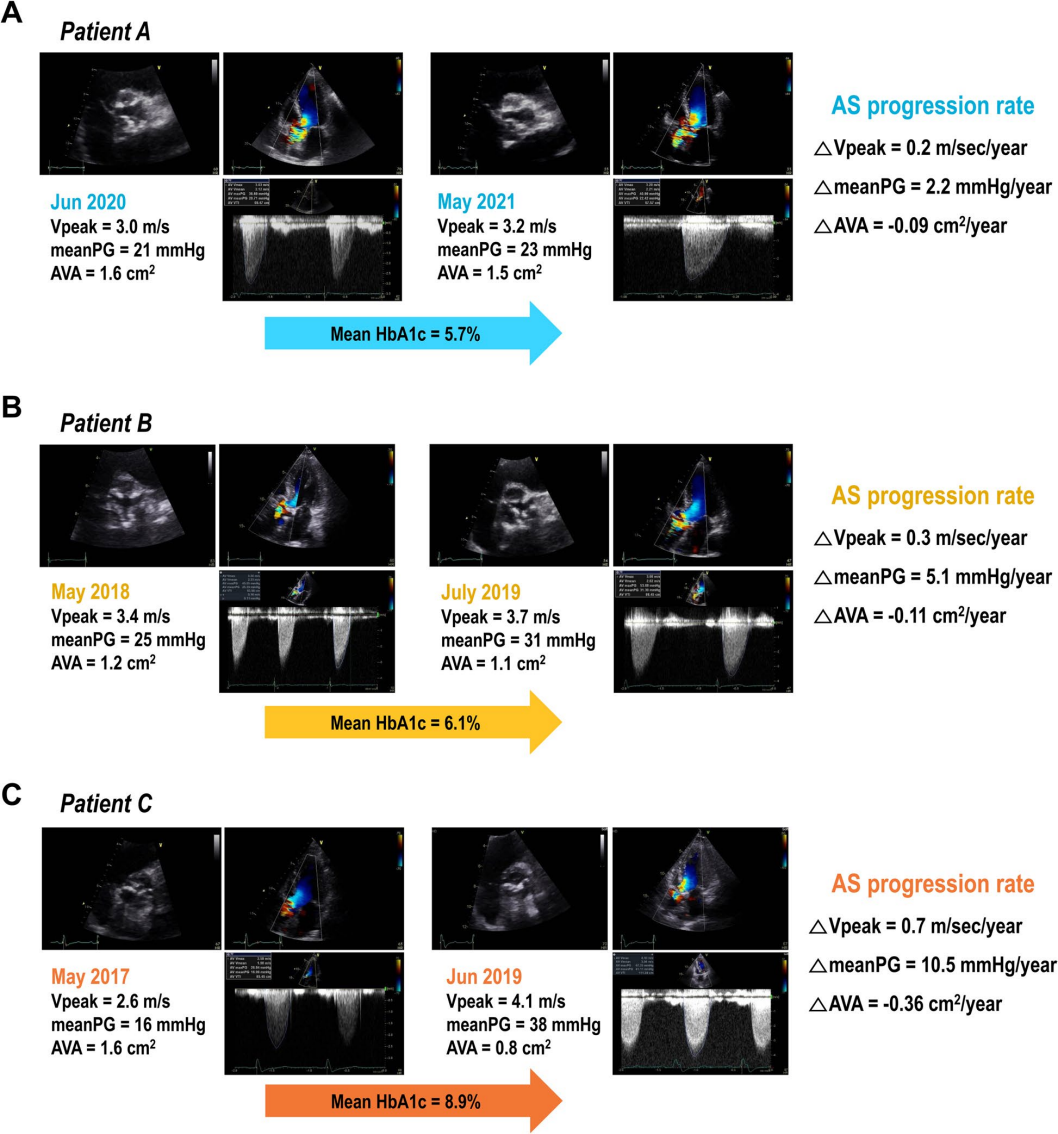
Representative cases. **A**: *Patient A*, without diabetes, had low HbA1c levels during follow-up (mean HbA1c, 5.7%) and showed a slow progression of AS (△Vpeak/year, 0.2 m/sec/year; △meanPG, 2.2 mmHg/year; △AVA, -0.09 cm^2^/year). **B**: *Patient B* was diagnosed as having diabetes, and maintained a tight glycemic control (mean HbA1c, 6.1%). During follow-up, the progression of AS was modest (△Vpeak/year, 0.3 m/sec/year; △meanPG, 5.1 mmHg/year; △AVA, -0.11 cm^2^/year). **C**: *Patient C*, in whom the glycemic control was poor (mean HbA1c, 8.9%), showed an accelerated AS progression during follow-up (△Vpeak/year, 0.7 m/sec/year; △meanPG, 10.5 mmHg/year; △AVA, -0.36 cm.^2^/year)

In the present study, we compared the AS progression rate based on echocardiographic parameters between patients without DM, with well-controlled DM, and with poorly-controlled DM. The mean HbA1c level during the study period was used as the indicator of glycemic control, which enabled the direct assessment of the association between the degree of glycemic control and the AS progression rate. Compared to that in patients without DM, AS progression was accelerated in patients with DM, and the progression was accelerated to a greater degree in those with poor glycemic control than in those with well-controlled DM (representative cases shown in Fig. 4). These findings emphasize the importance of strict glycemic control in patients with mild to moderate AS, in order to prevent the development of severe AS and the resultant invasive procedures, as well as a poor prognosis. Additionally, these findings suggest that the benefits of strict glycemic control inpatients with DM are not limited to the prevention of coronary events, but also include the attenuation of AS progression.

Furthermore, the potential benefit of strict glycemic control was consistently observed across the entire range of baseline AS severity in our study population. The present study results confirm previous studies that showed a more rapid progression rate in those with higher baseline Vpeak. Additionally, we newly showed that the degree of glycemic control affected the AS progression rates regardless of the baseline AS severity, suggesting that strict glycemic control would have consistent benefits in patients with mild to moderate AS.

The present study has several limitations. First, it was a retrospective cohort study, and thus, the causal relationship between glycemic control and AS progression requires further confirmation in a clinical trial. However, it would be ethically unacceptable to leave the poorly-controlled diabetic patients without appropriate glycemic control in a prospective study. Thus, the retrospective study design was partly inevitable to assess the impact of poor glycemic control on the progression of AS. Instead, we performed thorough CDM-based analyses, and successfully demonstrated the impact of glycemic control on the progression of AS. Second, we could not assess differences in AS progression rate according to the class or dosage of antidiabetic drugs, because the antidiabetic drugs prescribed in the study population were frequently adjusted during follow-up. Given the potential effects of the antidiabetic drug class on the AS progression rate [17], further studies with larger sample size or clinical trials are warranted. Third, our study focused on the changes in echocardiographic parameters, but not AV calcification as assessed by computed tomography, or AV inflammation as assessed by nuclear imaging. Finally, we included patients with mild or moderate AS, and therefore, our findings may not be applicable to those with AV calcification without stenosis, or those with advanced severe AS. However, considering the pathophysiology of AS progression, strict diabetic control might be beneficial in those with degenerative AV changes, even before the development of overt AS. In patients with severe AS, the benefits of strict glycemic control might not include the prevention of AS progression; however, the benefit in terms the prevention of other cardiovascular diseases is still valid.

## Conclusion

In patients with mild or moderate AS, the presence of DM is significantly associated with accelerated AS progression. Further, the progression of AS is accelerated to a greater degree in diabetic patients with poor glycemic control than in those with well-controlled DM.

## Supplementary Information

**Additional file 1:** **Supplementary Table S1. **Codes and definitions. **Supplementary Table S2. **Univariable linear regression analysis for the predictors of AS progression rate. **Supplementary Table S3. **Univariable logistic regression analysis for the predictors of accelerated AS progression.

## Data Availability

The de-identified data that support the findings of this study are available from the corresponding author upon reasonable request and appropriate permission from the institutional review board.

## Abbreviations

AS: Aortic stenosis
AGEs: Advanced glycation end products
AV: Aortic valve
AVA: Aortic valve area
CDM: Common data model
CI: Confidence interval
DM: Diabetes mellitus
HER: Electronic health record
HbA1c: Glycated hemoglobin
IQR: Interquartile range
OR: Odds ratio
Vpeak: Aortic valve maximal velocity
meanPG: Mean pressure gradient across the aortic valve
