# Supplementary material for: Impact of glycemic control on the progression of aortic stenosis: a single-center cohort study using a common data model
Source: BMC Endocr Disord. 2023 Jul 10;23:143. doi: 10.1186/s12902-023-01403-5 (PMC10331980; doi:10.1186/s12902-023-01403-5)
Supplement: Supplementary file 1 — Additional file 1: Supplementary Table S1. Codes and definitions. Supplementary Table S2. Univariable linear regression analysis for the predictors of AS progression rate. Supplementary Table S3. Univariable logistic regression analysis for the predictors of accelerated AS progression. [file 12902_2023_1403_MOESM1_ESM.docx]

**Supplementary materials**

**Supplementary Table S1. Codes and definitions**

| **Type** | **Item** | **Concept ID** | **Concept name** | **Domain** | **Vocabulary** | **Excluded** | **Descendants** | **Mapped** |
| --- | --- | --- | --- | --- | --- | --- | --- | --- |
| **Cohort** | AV Vmax | 3028570 | Aortic valve Peak systolic flow by US.Doppler | Measurement | LOINC | NO | NO | NO |
|  | Aortic valve replacement | 4095407 | Replacement of aortic valve | Procedure | SNOMED | NO | NO | NO |
|  | Aortic valve replacement | 4339824 | Aortic valve replacement and replacement of ascending aorta | Procedure | SNOMED | NO | NO | NO |
|  | Aortic valve replacement | 35609791 | TAVI - transcatheter aortic valve implantation | Procedure | SNOMED | NO | NO | NO |
| **Comorbidities** | Coronary artery disease | 312327 | Acute myocardial infarction | Condition | SNOMED | NO | NO | NO |
|  | Coronary artery disease | 43021858 | Arteriosclerosis of autologous coronary artery bypass graft | Condition | SNOMED | NO | NO | NO |
|  | Coronary artery disease | 315286 | Chronic ischemic heart disease | Condition | SNOMED | NO | NO | NO |
|  | Coronary artery disease | 315296 | Preinfarction syndrome | Condition | SNOMED | NO | NO | NO |
|  | Coronary artery disease | 315830 | Prinzmetal angina | Condition | SNOMED | NO | NO | NO |
|  | Coronary artery disease | 316995 | Coronary occlusion | Condition | SNOMED | NO | NO | NO |
|  | Coronary artery disease | 317576 | Coronary arteriosclerosis | Condition | SNOMED | NO | NO | NO |
|  | Coronary artery disease | 318443 | Arteriosclerotic vascular disease | Condition | SNOMED | NO | NO | NO |
|  | Coronary artery disease | 319038 | Postmyocardial infarction syndrome | Condition | SNOMED | NO | NO | NO |
|  | Coronary artery disease | 319039 | Acute posterior myocardial infarction | Condition | SNOMED | NO | NO | NO |
|  | Coronary artery disease | 319844 | Acute ischemic heart disease | Condition | SNOMED | NO | NO | NO |
|  | Coronary artery disease | 434376 | Acute myocardial infarction of anterior wall | Condition | SNOMED | NO | NO | NO |
|  | Coronary artery disease | 435561 | Certain current complications following acute myocardial infarction | Condition | SNOMED | NO | NO | NO |
|  | Coronary artery disease | 436706 | Acute myocardial infarction of lateral wall | Condition | SNOMED | NO | NO | NO |
|  | Coronary artery disease | 438168 | Aneurysm of heart | Condition | SNOMED | NO | NO | NO |
|  | Coronary artery disease | 438170 | Acute myocardial infarction of inferior wall | Condition | SNOMED | NO | NO | NO |
|  | Coronary artery disease | 444406 | Acute subendocardial infarction | Condition | SNOMED | NO | NO | NO |
|  | Coronary artery disease | 4078431 | Ischemic reperfusion injury | Condition | SNOMED | NO | NO | NO |
|  | Coronary artery disease | 4094054 | Stunned myocardium | Condition | SNOMED | NO | NO | NO |
|  | Coronary artery disease | 4094055 | Hibernating myocardium | Condition | SNOMED | NO | NO | NO |
|  | Coronary artery disease | 4108215 | Coronary thrombosis not resulting in myocardial infarction | Condition | SNOMED | NO | NO | NO |
|  | Coronary artery disease | 4108217 | Subsequent myocardial infarction | Condition | SNOMED | NO | NO | NO |
|  | Coronary artery disease | 4108218 | Subsequent myocardial infarction of inferior wall | Condition | SNOMED | NO | NO | NO |
|  | Coronary artery disease | 4108219 | Rupture of chordae tendinae due to and following acute myocardial infarction | Condition | SNOMED | NO | NO | NO |
|  | Coronary artery disease | 4108220 | Rupture of papillary muscle as current complication following acute myocardial infarction | Condition | SNOMED | NO | NO | NO |
|  | Coronary artery disease | 4108673 | Double coronary vessel disease | Condition | SNOMED | NO | NO | NO |
|  | Coronary artery disease | 4108677 | Subsequent myocardial infarction of anterior wall | Condition | SNOMED | NO | NO | NO |
|  | Coronary artery disease | 4108680 | Thrombosis of atrium, auricular appendage, and ventricle due to and following acute myocardial infarction | Condition | SNOMED | NO | NO | NO |
|  | Coronary artery disease | 4110961 | Generalized ischemic myocardial dysfunction | Condition | SNOMED | NO | NO | NO |
|  | Coronary artery disease | 4111393 | Single coronary vessel disease | Condition | SNOMED | NO | NO | NO |
|  | Coronary artery disease | 4116486 | Exercise-induced angina | Condition | SNOMED | NO | NO | NO |
|  | Coronary artery disease | 4119597 | Post-infarction hemopericardium | Condition | SNOMED | NO | NO | NO |
|  | Coronary artery disease | 4119942 | Stable angina | Condition | SNOMED | NO | NO | NO |
|  | Coronary artery disease | 4119949 | Old anterior myocardial infarction | Condition | SNOMED | NO | NO | NO |
|  | Coronary artery disease | 4119950 | Old lateral myocardial infarction | Condition | SNOMED | NO | NO | NO |
|  | Coronary artery disease | 4119952 | Cardiac syndrome X | Condition | SNOMED | NO | NO | NO |
|  | Coronary artery disease | 4119953 | Post-infarction ventricular septal defect | Condition | SNOMED | NO | NO | NO |
|  | Coronary artery disease | 4121467 | Old inferior myocardial infarction | Condition | SNOMED | NO | NO | NO |
|  | Coronary artery disease | 4121468 | Old posterior myocardial infarction | Condition | SNOMED | NO | NO | NO |
|  | Coronary artery disease | 4121477 | Post-infarction pericarditis | Condition | SNOMED | NO | NO | NO |
|  | Coronary artery disease | 4124682 | Triple vessel disease of the heart | Condition | SNOMED | NO | NO | NO |
|  | Coronary artery disease | 4124683 | Silent myocardial ischemia | Condition | SNOMED | NO | NO | NO |
|  | Coronary artery disease | 4124687 | Cardiac rupture due to and following acute myocardial infarction | Condition | SNOMED | NO | NO | NO |
|  | Coronary artery disease | 4126801 | Acute Q wave myocardial infarction | Condition | SNOMED | NO | NO | NO |
|  | Coronary artery disease | 4145721 | Acute non-Q wave infarction | Condition | SNOMED | NO | NO | NO |
|  | Coronary artery disease | 4155963 | Atypical angina | Condition | SNOMED | NO | NO | NO |
|  | Coronary artery disease | 4182190 | Left ventricular aneurysm | Condition | SNOMED | NO | NO | NO |
|  | Coronary artery disease | 4185932 | Ischemic heart disease | Condition | SNOMED | NO | NO | NO |
|  | Coronary artery disease | 4186397 | Myocardial ischemia | Condition | SNOMED | NO | NO | NO |
|  | Coronary artery disease | 4198141 | Post infarct angina | Condition | SNOMED | NO | NO | NO |
|  | Coronary artery disease | 4215140 | Acute coronary syndrome | Condition | SNOMED | NO | NO | NO |
|  | Coronary artery disease | 4270024 | Acute non-ST segment elevation myocardial infarction | Condition | SNOMED | NO | NO | NO |
|  | Coronary artery disease | 4315010 | Dysfunction of papillary muscle | Condition | SNOMED | NO | NO | NO |
|  | Coronary artery disease | 4329847 | Myocardial infarction | Condition | SNOMED | NO | NO | NO |
|  | Coronary artery disease | 40481919 | Coronary atherosclerosis | Condition | SNOMED | NO | NO | NO |
|  | Coronary artery disease | 40482655 | Arteriosclerosis of nonautologous coronary artery bypass graft | Condition | SNOMED | NO | NO | NO |
|  | Coronary artery disease | 42872402 | Coronary arteriosclerosis in native artery | Condition | SNOMED | NO | NO | NO |
|  | Coronary artery disease | 314666 | Old myocardial infarction | Condition | SNOMED | NO | NO | NO |
|  | Atrial fibrillation | 4232697 | Persistent atrial fibrillation | Condition | SNOMED | NO | NO | NO |
|  | Atrial fibrillation | 313217 | Atrial fibrillation | Condition | SNOMED | NO | NO | NO |
|  | Atrial fibrillation | 4108832 | Atrial fibrillation and flutter | Condition | SNOMED | NO | NO | NO |
|  | Atrial fibrillation | 314665 | Atrial flutter | Condition | SNOMED | NO | NO | NO |
|  | Atrial fibrillation | 4141360 | Chronic atrial fibrillation | Condition | SNOMED | NO | NO | NO |
|  | Atrial fibrillation | 4154290 | Paroxysmal atrial fibrillation | Condition | SNOMED | NO | NO | NO |
|  | Atrial fibrillation | 4146580 | Paroxysmal atrial flutter | Condition | SNOMED | NO | NO | NO |
|  | CKD | 192359 | Renal failure syndrome | Condition | SNOMED | NO | NO | NO |
|  | CKD | 193782 | End-stage renal disease | Condition | SNOMED | NO | NO | NO |
|  | CKD | 4137220 | Diabetic glomerulonephritis | Condition | SNOMED | NO | NO | NO |
|  | CKD | 4059452 | Glomerular disease | Condition | SNOMED | NO | NO | NO |
|  | CKD | 4030520 | End stage renal failure on dialysis | Condition | SNOMED | NO | NO | NO |
|  | CKD | 4028050 | Sclerosing glomerulonephritis | Condition | SNOMED | NO | NO | NO |
|  | CKD | 4008236 | Absent renal function | Condition | SNOMED | NO | NO | NO |
|  | CKD | 443731 | Renal disorder due to type 2 diabetes mellitus | Condition | SNOMED | NO | NO | NO |
|  | CKD | 443612 | Chronic kidney disease stage 4 | Condition | SNOMED | NO | NO | NO |
|  | CKD | 443611 | Chronic kidney disease stage 5 | Condition | SNOMED | NO | NO | NO |
|  | CKD | 443601 | Chronic kidney disease stage 2 | Condition | SNOMED | NO | NO | NO |
|  | CKD | 443597 | Chronic kidney disease stage 3 | Condition | SNOMED | NO | NO | NO |
|  | CKD | 201826 | Type 2 diabetes mellitus | Condition | SNOMED | NO | NO | NO |
|  | CKD | 201254 | Type 1 diabetes mellitus | Condition | SNOMED | NO | NO | NO |
|  | CKD | 200687 | Renal disorder due to type 1 diabetes mellitus | Condition | SNOMED | NO | NO | NO |
|  | CKD | 198185 | Chronic renal failure | Condition | SNOMED | NO | NO | NO |
|  | CKD | 46271022 | Chronic kidney disease | Condition | SNOMED | NO | NO | NO |
|  | Diabetes mellitus | 192279 | Disorder of kidney due to diabetes mellitus | Condition | SNOMED | NO | NO | NO |
|  | Diabetes mellitus | 195771 | Secondary diabetes mellitus | Condition | SNOMED | NO | NO | NO |
|  | Diabetes mellitus | 200687 | Renal disorder due to type 1 diabetes mellitus | Condition | SNOMED | NO | NO | NO |
|  | Diabetes mellitus | 201254 | Type 1 diabetes mellitus | Condition | SNOMED | NO | NO | NO |
|  | Diabetes mellitus | 201820 | Diabetes mellitus | Condition | SNOMED | NO | NO | NO |
|  | Diabetes mellitus | 201826 | Type 2 diabetes mellitus | Condition | SNOMED | NO | NO | NO |
|  | Diabetes mellitus | 318712 | Peripheral circulatory disorder due to type 1 diabetes mellitus | Condition | SNOMED | NO | NO | NO |
|  | Diabetes mellitus | 321822 | Peripheral vascular disorder due to diabetes mellitus | Condition | SNOMED | NO | NO | NO |
|  | Diabetes mellitus | 373999 | Diabetic oculopathy associated with type 1 diabetes mellitus | Condition | SNOMED | NO | NO | NO |
|  | Diabetes mellitus | 376065 | Disorder of nervous system due to type 2 diabetes mellitus | Condition | SNOMED | NO | NO | NO |
|  | Diabetes mellitus | 376112 | Polyneuropathy due to diabetes mellitus | Condition | SNOMED | NO | NO | NO |
|  | Diabetes mellitus | 377821 | Disorder of nervous system due to type 1 diabetes mellitus | Condition | SNOMED | NO | NO | NO |
|  | Diabetes mellitus | 435216 | Disorder due to type 1 diabetes mellitus | Condition | SNOMED | NO | NO | NO |
|  | Diabetes mellitus | 442793 | Complication due to diabetes mellitus | Condition | SNOMED | NO | NO | NO |
|  | Diabetes mellitus | 443727 | Diabetic ketoacidosis | Condition | SNOMED | NO | NO | NO |
|  | Diabetes mellitus | 443729 | Peripheral circulatory disorder due to type 2 diabetes mellitus | Condition | SNOMED | NO | NO | NO |
|  | Diabetes mellitus | 443730 | Disorder of nervous system due to diabetes mellitus | Condition | SNOMED | NO | NO | NO |
|  | Diabetes mellitus | 443731 | Renal disorder due to type 2 diabetes mellitus | Condition | SNOMED | NO | NO | NO |
|  | Diabetes mellitus | 443732 | Disorder due to type 2 diabetes mellitus | Condition | SNOMED | NO | NO | NO |
|  | Diabetes mellitus | 443733 | Disorder of eye due to type 2 diabetes mellitus | Condition | SNOMED | NO | NO | NO |
|  | Diabetes mellitus | 443735 | Coma due to diabetes mellitus | Condition | SNOMED | NO | NO | NO |
|  | Diabetes mellitus | 443767 | Disorder of eye due to diabetes mellitus | Condition | SNOMED | NO | NO | NO |
|  | Diabetes mellitus | 4008576 | Diabetes mellitus without complication | Condition | SNOMED | NO | NO | NO |
|  | Diabetes mellitus | 4019513 | Brittle diabetes mellitus | Condition | SNOMED | NO | NO | NO |
|  | Diabetes mellitus | 4029422 | Hypoglycemic event due to diabetes | Condition | SNOMED | NO | NO | NO |
|  | Diabetes mellitus | 4030664 | Proteinuric nephropathy due to diabetes mellitus | Condition | SNOMED | NO | NO | NO |
|  | Diabetes mellitus | 4034960 | Secondary endocrine diabetes mellitus | Condition | SNOMED | NO | NO | NO |
|  | Diabetes mellitus | 4034969 | Drug-induced hypoglycemia | Condition | SNOMED | NO | NO | NO |
|  | Diabetes mellitus | 4044391 | Neuropathy due to diabetes mellitus | Condition | SNOMED | NO | NO | NO |
|  | Diabetes mellitus | 4048028 | Diabetic mononeuropathy | Condition | SNOMED | NO | NO | NO |
|  | Diabetes mellitus | 4087682 | Diabetic foot | Condition | SNOMED | NO | NO | NO |
|  | Diabetes mellitus | 4095288 | Ketoacidotic coma due to diabetes mellitus | Condition | SNOMED | NO | NO | NO |
|  | Diabetes mellitus | 4096804 | Drug-induced hypoglycemia without coma | Condition | SNOMED | NO | NO | NO |
|  | Diabetes mellitus | 4099216 | Multiple complications due to type 2 diabetes mellitus | Condition | SNOMED | NO | NO | NO |
|  | Diabetes mellitus | 4099334 | Steroid-induced diabetes | Condition | SNOMED | NO | NO | NO |
|  | Diabetes mellitus | 4111658 | Congenital generalized lipodystrophy | Condition | SNOMED | NO | NO | NO |
|  | Diabetes mellitus | 4114427 | Neuropathic arthropathy due to diabetes mellitus | Condition | SNOMED | NO | NO | NO |
|  | Diabetes mellitus | 4128221 | Microalbuminuric diabetic nephropathy | Condition | SNOMED | NO | NO | NO |
|  | Diabetes mellitus | 4129225 | Motor polyneuropathy due to diabetes mellitus | Condition | SNOMED | NO | NO | NO |
|  | Diabetes mellitus | 4131117 | Sensory neuropathy due to diabetes mellitus | Condition | SNOMED | NO | NO | NO |
|  | Diabetes mellitus | 4131908 | Peripheral angiopathy due to diabetes mellitus | Condition | SNOMED | NO | NO | NO |
|  | Diabetes mellitus | 4147719 | Hyperosmolar non-ketotic state due to diabetes mellitus | Condition | SNOMED | NO | NO | NO |
|  | Diabetes mellitus | 4151281 | Type 1 diabetes mellitus with hypoglycemic coma | Condition | SNOMED | NO | NO | NO |
|  | Diabetes mellitus | 4151453 | Diabetic optic papillopathy | Condition | SNOMED | NO | NO | NO |
|  | Diabetes mellitus | 4159742 | Diabetic foot ulcer | Condition | SNOMED | NO | NO | NO |
|  | Diabetes mellitus | 4174977 | Retinopathy due to diabetes mellitus | Condition | SNOMED | NO | NO | NO |
|  | Diabetes mellitus | 4175440 | Autonomic neuropathy due to diabetes mellitus | Condition | SNOMED | NO | NO | NO |
|  | Diabetes mellitus | 4178452 | Diabetes mellitus associated with pancreatic disease | Condition | SNOMED | NO | NO | NO |
|  | Diabetes mellitus | 4189418 | Radiculoplexus neuropathy due to diabetes mellitus | Condition | SNOMED | NO | NO | NO |
|  | Diabetes mellitus | 4193704 | Type 2 diabetes mellitus without complication | Condition | SNOMED | NO | NO | NO |
|  | Diabetes mellitus | 4198296 | Type 2 diabetes mellitus with neuropathic arthropathy | Condition | SNOMED | NO | NO | NO |
|  | Diabetes mellitus | 4202383 | Drug-induced diabetes mellitus | Condition | SNOMED | NO | NO | NO |
|  | Diabetes mellitus | 4224709 | Multiple complications due to type 1 diabetes mellitus | Condition | SNOMED | NO | NO | NO |
|  | Diabetes mellitus | 4226238 | Hyperosmolar coma due to diabetes mellitus | Condition | SNOMED | NO | NO | NO |
|  | Diabetes mellitus | 4230254 | Type 2 diabetes mellitus in nonobese | Condition | SNOMED | NO | NO | NO |
|  | Diabetes mellitus | 4245270 | Diabetes mellitus associated with genetic syndrome | Condition | SNOMED | NO | NO | NO |
|  | Diabetes mellitus | 4262282 | Diabetic mononeuropathy multiplex | Condition | SNOMED | NO | NO | NO |
|  | Diabetes mellitus | 4304377 | Type 2 diabetes mellitus in obese | Condition | SNOMED | NO | NO | NO |
|  | Diabetes mellitus | 4322638 | Diabetes mellitus AND insipidus with optic atrophy AND deafness | Condition | SNOMED | NO | NO | NO |
|  | Diabetes mellitus | 4327944 | Malnutrition related diabetes mellitus | Condition | SNOMED | NO | NO | NO |
|  | Diabetes mellitus | 36714116 | Hypoglycemic coma due to type 2 diabetes mellitus | Condition | SNOMED | NO | NO | NO |
|  | Diabetes mellitus | 37017430 | Gastroparesis due to diabetes mellitus | Condition | SNOMED | NO | NO | NO |
|  | Diabetes mellitus | 40482883 | Posttransplant diabetes mellitus | Condition | SNOMED | NO | NO | NO |
|  | Diabetes mellitus | 43530690 | Foot ulcer due to type 2 diabetes mellitus | Condition | SNOMED | NO | NO | NO |
|  | Diabetes mellitus | 43531006 | Maturity onset diabetes of the young, type 1 | Condition | SNOMED | NO | NO | NO |
|  | Diabetes mellitus | 43531640 | Maturity-onset diabetes of the young | Condition | SNOMED | NO | NO | NO |
|  | Diabetes mellitus | 45757507 | Ulcer of foot due to type 1 diabetes mellitus | Condition | SNOMED | NO | NO | NO |
|  | Diabetes mellitus | 45769830 | Neuropathic arthropathy due to type 1 diabetes mellitus | Condition | SNOMED | NO | NO | NO |
|  | Dyslipidemia | 432867 | Hyperlipidemia | Condition | SNOMED | NO | NO | NO |
|  | Dyslipidemia | 4292079 | Hyperlipoproteinemia | Condition | SNOMED | NO | NO | NO |
|  | Dyslipidemia | 437530 | Disorder of lipid metabolism | Condition | SNOMED | NO | NO | NO |
|  | Dyslipidemia | 4029261 | Fredrickson type IV hyperlipoproteinemia | Condition | SNOMED | NO | NO | NO |
|  | Dyslipidemia | 4029262 | Familial lipoprotein lipase deficiency | Condition | SNOMED | NO | NO | NO |
|  | Dyslipidemia | 4029263 | Primary combined hyperlipidemia | Condition | SNOMED | NO | NO | NO |
|  | Dyslipidemia | 4029305 | Hypercholesterolemia | Condition | SNOMED | NO | NO | NO |
|  | Dyslipidemia | 4036109 | Familial hypoalphalipoproteinemia | Condition | SNOMED | NO | NO | NO |
|  | Dyslipidemia | 4079876 | Familial combined hyperlipidemia | Condition | SNOMED | NO | NO | NO |
|  | Dyslipidemia | 4098481 | Hyperbetalipoproteinemia | Condition | SNOMED | NO | NO | NO |
|  | Dyslipidemia | 4120314 | Hypertriglyceridemia | Condition | SNOMED | NO | NO | NO |
|  | Dyslipidemia | 4134862 | Familial hypercholesterolemia | Condition | SNOMED | NO | NO | NO |
|  | Dyslipidemia | 4144529 | Familial type 5 hyperlipoproteinemia | Condition | SNOMED | NO | NO | NO |
|  | Dyslipidemia | 4159131 | Dyslipidemia | Condition | SNOMED | NO | NO | NO |
|  | Dyslipidemia | 4162681 | Familial type 3 hyperlipoproteinemia | Condition | SNOMED | NO | NO | NO |
|  | Dyslipidemia | 4170226 | Disorder of lipoprotein AND/OR lipid metabolism | Condition | SNOMED | NO | NO | NO |
|  | Dyslipidemia | 4220010 | Fredrickson type IIa hyperlipoproteinemia | Condition | SNOMED | NO | NO | NO |
|  | Dyslipidemia | 437521 | Familial hyperchylomicronemia | Condition | SNOMED | NO | NO | NO |
|  | Heart failure | 4124706 | Myocardial dysfunction | Condition | SNOMED | NO | NO | NO |
|  | Heart failure | 4181684 | Right ventricular function - finding | Condition | SNOMED | NO | NO | NO |
|  | Heart failure | 4235646 | Acute cardiac pulmonary edema | Condition | SNOMED | NO | NO | NO |
|  | Heart failure | 4323898 | Left ventricular cardiac dysfunction | Condition | SNOMED | NO | NO | NO |
|  | Heart failure | 439846 | Left heart failure | Condition | SNOMED | NO | NO | NO |
|  | Heart failure | 4108153 | Right ventricular function | Condition | SNOMED | NO | NO | NO |
|  | Heart failure | 4273632 | Right ventricular failure | Condition | SNOMED | NO | NO | NO |
|  | Heart failure | 138255 | Abnormal cardiovascular function | Condition | SNOMED | NO | NO | NO |
|  | Heart failure | 316139 | Heart failure | Condition | SNOMED | NO | NO | NO |
|  | Heart failure | 4047088 | Left ventricular systolic dysfunction | Condition | SNOMED | NO | NO | NO |
|  | Heart failure | 319835 | Congestive heart failure | Condition | SNOMED | NO | NO | NO |
|  | Heart failure | 4134890 | Right heart failure | Condition | SNOMED | NO | NO | NO |
|  | Hypertension | 442604 | Hypertensive heart disease | Condition | SNOMED | NO | NO | NO |
|  | Hypertension | 443771 | Renal hypertension | Condition | SNOMED | NO | NO | NO |
|  | Hypertension | 4028741 | Benign hypertension | Condition | SNOMED | NO | NO | NO |
|  | Hypertension | 4108213 | Hypertension secondary to drug | Condition | SNOMED | NO | NO | NO |
|  | Hypertension | 4110948 | Hypertension secondary to endocrine disorder | Condition | SNOMED | NO | NO | NO |
|  | Hypertension | 4289933 | Malignant hypertension | Condition | SNOMED | NO | NO | NO |
|  | Hypertension | 439697 | Hypertensive renal disease with renal failure | Condition | SNOMED | NO | NO | NO |
|  | Hypertension | 439696 | Hypertensive heart and renal disease with (congestive) heart failure | Condition | SNOMED | NO | NO | NO |
|  | Hypertension | 439695 | Hypertensive heart and renal disease with renal failure | Condition | SNOMED | NO | NO | NO |
|  | Hypertension | 439694 | Hypertensive heart and renal disease with both (congestive) heart failure and renal failure | Condition | SNOMED | NO | NO | NO |
|  | Hypertension | 378774 | Moyamoya disease | Condition | SNOMED | NO | NO | NO |
|  | Hypertension | 320128 | Essential hypertension | Condition | SNOMED | NO | NO | NO |
|  | Hypertension | 319826 | Secondary hypertension | Condition | SNOMED | NO | NO | NO |
|  | Hypertension | 319034 | Hypertensive heart disease without congestive heart failure | Condition | SNOMED | NO | NO | NO |
|  | Hypertension | 317895 | Renovascular hypertension | Condition | SNOMED | NO | NO | NO |
|  | Hypertension | 316866 | Hypertensive disorder | Condition | SNOMED | NO | NO | NO |
|  | Hypertension | 314378 | Hypertensive heart disease with congestive heart failure | Condition | SNOMED | NO | NO | NO |
|  | Hypertension | 195556 | Hypertensive heart AND renal disease | Condition | SNOMED | NO | NO | NO |
|  | Hypertension | 201313 | Hypertensive renal disease | Condition | SNOMED | NO | NO | NO |
| **Echocardiographic**  **findings** | LVEDD | 3014726 | Left ventricle [Length] Minor axis.diastole US.M-mode+Measured | Measurement | LOINC | NO | NO | NO |
|  | LVEDV | 3012854 | Left ventricular End-diastolic volume by US.2D+Calculated by biplane ellipse method | Measurement | LOINC | NO | NO | NO |
|  | LVESD | 3015876 | Left ventricle [Length] Short axis during systole US.M-mode+Measured | Measurement | LOINC | NO | NO | NO |
|  | LVESV | 3024038 | Left ventricular End-systolic volume by US.2D+Calculated by biplane ellipse method | Measurement | LOINC | NO | NO | NO |
|  | LVMI | 46236336 | Left ventricular Myocardial mass/Body surface area [Mass/Area] by US.M-mode+Calculated by cube method | Measurement | LOINC | NO | NO | NO |
|  | Vpeak | 3028570 | Aortic valve Peak systolic flow by US.Doppler | Measurement | LOINC | NO | NO | NO |
|  | MSPG | 21493999 | Aortic valve Mean systole pressure gradient by US.Doppler+Calculated by simplified Bernoulli | Measurement | LOINC | NO | NO | NO |
|  | AVA(by Cont.) | 3003445 | Aortic valve Orifice area by US.continuity.VTI+Diameter | Measurement | LOINC | NO | NO | NO |
|  | Height | 3036277 | Body height | Measurement | LOINC | NO | NO | NO |
|  | Weight | 3025315 | Body weight | Measurement | LOINC | NO | NO | NO |
|  | SBP | 3004249 | Systolic blood pressure | Measurement | LOINC | NO | NO | NO |
|  | DBP | 3012888 | Diastolic blood pressure | Measurement | LOINC | NO | NO | NO |
|  | HR | 3027018 | Heart rate | Measurement | LOINC | NO | NO | NO |
| **Laboratory**  **findings** | Hb (g/dL) | 3000963 | Hemoglobin [Mass/volume] in Blood | Measurement | LOINC | NO | NO | NO |
|  | Serum Cr (mg/dL) | 3016723 | Creatinine [Mass/volume] in Serum or Plasma | Measurement | LOINC | NO | NO | NO |
|  | GFR | 2000000118 | EPI-CKD eGFR, Cr-based | Measurement | SNUBH generated | NO | NO | NO |
|  | Fasting glucose (mg/dL) | 44816672 | Glucose [Mass/volume] in Serum, Plasma or Blood | Measurement | LOINC | NO | NO | NO |
|  | HbA1c (%) | 3005673 | Hemoglobin A1c/Hemoglobin.total in Blood by HPLC | Measurement | LOINC | NO | NO | NO |
|  | Total cholesterol (mg/dL) | 3027114 | Cholesterol [Mass/volume] in Serum or Plasma | Measurement | LOINC | NO | NO | NO |
|  | Triglyceride (mg/dL) | 3022192 | Triglyceride [Mass/volume] in Serum or Plasma | Measurement | LOINC | NO | NO | NO |
|  | HDL cholesterol (mg/dL) | 3007070 | Cholesterol in HDL [Mass/volume] in Serum or Plasma | Measurement | LOINC | NO | NO | NO |
| **Medication** | Alpha_glucosidase_inhibitors | 43009032 | voglibose | Drug | RxNorm Extension | NO | NO | NO |
|  | Alpha_glucosidase_inhibitors | 1529331 | acarbose | Drug | RxNorm | NO | NO | NO |
|  | Beta | 902427 | timolol | Drug | RxNorm | NO | NO | NO |
|  | Beta | 43009042 | bevantolol hydrochloride | Drug | RxNorm Extension | NO | NO | NO |
|  | Beta | 1314002 | atenolol | Drug | RxNorm | NO | NO | NO |
|  | Beta | 1314577 | nebivolol | Drug | RxNorm | NO | NO | NO |
|  | Beta | 1319998 | acebutolol | Drug | RxNorm | NO | NO | NO |
|  | Beta | 1322081 | betaxolol | Drug | RxNorm | NO | NO | NO |
|  | Beta | 1327978 | penbutolol | Drug | RxNorm | NO | NO | NO |
|  | Beta | 1338005 | bisoprolol | Drug | RxNorm | NO | NO | NO |
|  | Beta | 1345858 | pindolol | Drug | RxNorm | NO | NO | NO |
|  | Beta | 1346823 | carvedilol | Drug | RxNorm | NO | NO | NO |
|  | Beta | 1353766 | propranolol | Drug | RxNorm | NO | NO | NO |
|  | Beta | 1370109 | sotalol | Drug | RxNorm | NO | NO | NO |
|  | Beta | 1386957 | labetalol | Drug | RxNorm | NO | NO | NO |
|  | Beta | 19018489 | bupranolol | Drug | RxNorm | NO | NO | NO |
|  | Beta | 19018640 | bopindolol | Drug | RxNorm | NO | NO | NO |
|  | Beta | 19024904 | oxprenolol | Drug | RxNorm | NO | NO | NO |
|  | Beta | 19049145 | celiprolol | Drug | RxNorm | NO | NO | NO |
|  | Beta | 19063575 | esmolol | Drug | RxNorm | NO | NO | NO |
|  | Beta | 19072028 | mepindolol | Drug | RxNorm | NO | NO | NO |
|  | Beta | 19081284 | alprenolol | Drug | RxNorm | NO | NO | NO |
|  | Beta | 19100435 | talinolol | Drug | RxNorm | NO | NO | NO |
|  | Beta | 19100451 | tertatolol | Drug | RxNorm | NO | NO | NO |
|  | Beta | 19135791 | practolol | Drug | RxNorm | NO | NO | NO |
|  | Beta | 35197852 | landiolol hydrochloride | Drug | RxNorm Extension | NO | NO | NO |
|  | Beta | 1313200 | nadolol | Drug | RxNorm | NO | NO | NO |
|  | CCB | 1307863 | verapamil | Drug | RxNorm | NO | NO | NO |
|  | CCB | 43009040 | benidipine hydrochloride | Drug | RxNorm Extension | NO | NO | NO |
|  | CCB | 1318853 | nifedipine | Drug | RxNorm | NO | NO | NO |
|  | CCB | 1319133 | nimodipine | Drug | RxNorm | NO | NO | NO |
|  | CCB | 1319751 | bepridil | Drug | RxNorm | NO | NO | NO |
|  | CCB | 1319880 | nisoldipine | Drug | RxNorm | NO | NO | NO |
|  | CCB | 1326012 | isradipine | Drug | RxNorm | NO | NO | NO |
|  | CCB | 1328165 | diltiazem | Drug | RxNorm | NO | NO | NO |
|  | CCB | 1332418 | amlodipine | Drug | RxNorm | NO | NO | NO |
|  | CCB | 1345141 | mibefradil | Drug | RxNorm | NO | NO | NO |
|  | CCB | 1353776 | felodipine | Drug | RxNorm | NO | NO | NO |
|  | CCB | 19004539 | lacidipine | Drug | RxNorm | NO | NO | NO |
|  | CCB | 19015802 | lercanidipine | Drug | RxNorm | NO | NO | NO |
|  | CCB | 19020061 | nitrendipine | Drug | RxNorm | NO | NO | NO |
|  | CCB | 19032359 | perhexiline | Drug | RxNorm | NO | NO | NO |
|  | CCB | 19053866 | fendiline | Drug | RxNorm | NO | NO | NO |
|  | CCB | 19057715 | gallopamil | Drug | RxNorm | NO | NO | NO |
|  | CCB | 19071995 | manidipine | Drug | RxNorm | NO | NO | NO |
|  | CCB | 19089969 | clevidipine | Drug | RxNorm | NO | NO | NO |
|  | CCB | 19102106 | mepirodipine | Drug | RxNorm | NO | NO | NO |
|  | CCB | 19113063 | nilvadipine | Drug | RxNorm | NO | NO | NO |
|  | CCB | 19124331 | lidoflazine | Drug | RxNorm | NO | NO | NO |
|  | CCB | 43009017 | cilnidipine | Drug | RxNorm Extension | NO | NO | NO |
|  | CCB | 1318137 | nicardipine | Drug | RxNorm | NO | NO | NO |
|  | DPP4 | 43013884 | alogliptin | Drug | RxNorm | NO | NO | NO |
|  | DPP4 | 43009051 | evogliptin | Drug | RxNorm Extension | NO | NO | NO |
|  | DPP4 | 43009089 | gemigliptin | Drug | RxNorm Extension | NO | NO | NO |
|  | DPP4 | 19122137 | vildagliptin | Drug | RxNorm | NO | NO | NO |
|  | DPP4 | 40166035 | saxagliptin | Drug | RxNorm | NO | NO | NO |
|  | DPP4 | 1580747 | sitagliptin | Drug | RxNorm | NO | NO | NO |
|  | DPP4 | 43009070 | teneligliptin | Drug | RxNorm Extension | NO | NO | NO |
|  | DPP4 | 40239216 | linagliptin | Drug | RxNorm | NO | NO | NO |
|  | GLP1 | 44506754 | lixisenatide | Drug | RxNorm | NO | NO | NO |
|  | GLP1 | 45774435 | dulaglutide | Drug | RxNorm | NO | NO | NO |
|  | GLP1 | 793143 | semaglutide | Drug | RxNorm | NO | NO | NO |
|  | GLP1 | 1583722 | exenatide | Drug | RxNorm | NO | NO | NO |
|  | GLP1 | 44816332 | albiglutide | Drug | RxNorm | NO | NO | NO |
|  | GLP1 | 40170911 | liraglutide | Drug | RxNorm | NO | NO | NO |
|  | Insulin | 21600713 | INSULINS AND ANALOGUES | Drug | ATC | NO | NO | NO |
|  | Insulin | 1531601 | insulin aspart protamine, human | Drug | RxNorm | NO | NO | NO |
|  | Insulin | 35602717 | insulin degludec | Drug | RxNorm | NO | NO | NO |
|  | Insulin | 1502905 | insulin glargine | Drug | RxNorm | NO | NO | NO |
|  | Insulin | 1544838 | insulin glulisine, human | Drug | RxNorm | NO | NO | NO |
|  | Insulin | 46221581 | insulin isophane | Drug | RxNorm | NO | NO | NO |
|  | Insulin | 1550023 | insulin lispro | Drug | RxNorm | NO | NO | NO |
|  | Insulin | 1513876 | insulin lispro protamine, human | Drug | RxNorm | NO | NO | NO |
|  | Insulin | 1596977 | insulin, regular, human | Drug | RxNorm | NO | NO | NO |
|  | Insulin | 1586346 | insulin, regular, pork | Drug | RxNorm | NO | NO | NO |
|  | Metformin | 1503297 | metformin | Drug | RxNorm | NO | NO | NO |
|  | NOAC | 45775372 | dabigatran | Drug | RxNorm | NO | NO | NO |
|  | NOAC | 40241331 | rivaroxaban | Drug | RxNorm | NO | NO | NO |
|  | NOAC | 45892847 | edoxaban | Drug | RxNorm | NO | NO | NO |
|  | NOAC | 43013024 | apixaban | Drug | RxNorm | NO | NO | NO |
|  | RAS | 1335471 | benazepril | Drug | RxNorm | NO | NO | NO |
|  | RAS | 1340128 | captopril | Drug | RxNorm | NO | NO | NO |
|  | RAS | 1341927 | enalapril | Drug | RxNorm | NO | NO | NO |
|  | RAS | 1342001 | enalaprilat | Drug | RxNorm | NO | NO | NO |
|  | RAS | 1342439 | trandolapril | Drug | RxNorm | NO | NO | NO |
|  | RAS | 1346686 | eprosartan | Drug | RxNorm | NO | NO | NO |
|  | RAS | 1347384 | irbesartan | Drug | RxNorm | NO | NO | NO |
|  | RAS | 1351557 | candesartan | Drug | RxNorm | NO | NO | NO |
|  | RAS | 1363749 | fosinopril | Drug | RxNorm | NO | NO | NO |
|  | RAS | 1367500 | losartan | Drug | RxNorm | NO | NO | NO |
|  | RAS | 1373225 | perindopril | Drug | RxNorm | NO | NO | NO |
|  | RAS | 19040051 | spirapril | Drug | RxNorm | NO | NO | NO |
|  | RAS | 19050216 | cilazapril | Drug | RxNorm | NO | NO | NO |
|  | RAS | 19102107 | zofenopril | Drug | RxNorm | NO | NO | NO |
|  | RAS | 19122327 | imidapril | Drug | RxNorm | NO | NO | NO |
|  | RAS | 36878917 | delapril hydrochloride | Drug | RxNorm Extension | NO | NO | NO |
|  | RAS | 40226742 | olmesartan | Drug | RxNorm | NO | NO | NO |
|  | RAS | 40235485 | azilsartan | Drug | RxNorm | NO | NO | NO |
|  | RAS | 43009001 | fimasartan potassium | Drug | RxNorm Extension | NO | NO | NO |
|  | RAS | 43009010 | temocapril hydrochloride | Drug | RxNorm Extension | NO | NO | NO |
|  | RAS | 1334456 | ramipril | Drug | RxNorm | NO | NO | NO |
|  | RAS | 1331235 | quinapril | Drug | RxNorm | NO | NO | NO |
|  | RAS | 1317640 | telmisartan | Drug | RxNorm | NO | NO | NO |
|  | RAS | 1310756 | moexipril | Drug | RxNorm | NO | NO | NO |
|  | RAS | 1308216 | lisinopril | Drug | RxNorm | NO | NO | NO |
|  | RAS | 1308842 | valsartan | Drug | RxNorm | NO | NO | NO |
|  | SGLT2 | 43009020 | ipragliflozin | Drug | RxNorm Extension | NO | NO | NO |
|  | SGLT2 | 45774751 | empagliflozin | Drug | RxNorm | NO | NO | NO |
|  | Sulfonylurea | 1597756 | glimepiride | Drug | RxNorm | NO | NO | NO |
|  | Sulfonylurea | 19059796 | gliclazide | Drug | RxNorm | NO | NO | NO |
|  | Thiazolidinediones | 1547504 | rosiglitazone | Drug | RxNorm | NO | NO | NO |
|  | Thiazolidinediones | 1525215 | pioglitazone | Drug | RxNorm | NO | NO | NO |
|  | Thiazolidinediones | 43009055 | lobeglitazone | Drug | RxNorm Extension | NO | NO | NO |
|  | antiplatelet | 40163718 | prasugrel | Drug | RxNorm | NO | NO | NO |
|  | antiplatelet | 1112807 | aspirin | Drug | RxNorm | NO | NO | NO |
|  | antiplatelet | 1322184 | clopidogrel | Drug | RxNorm | NO | NO | NO |
|  | antiplatelet | 40241186 | ticagrelor | Drug | RxNorm | NO | NO | NO |
|  | spironolactone | 970250 | spironolactone | Drug | RxNorm | NO | NO | NO |
|  | statin | 1592085 | lovastatin | Drug | RxNorm | NO | NO | NO |
|  | statin | 1539403 | simvastatin | Drug | RxNorm | NO | NO | NO |
|  | statin | 1510813 | rosuvastatin | Drug | RxNorm | NO | NO | NO |
|  | statin | 1545958 | atorvastatin | Drug | RxNorm | NO | NO | NO |
|  | statin | 40165636 | pitavastatin | Drug | RxNorm | NO | NO | NO |
|  | statin | 1592180 | cerivastatin | Drug | RxNorm | NO | NO | NO |
|  | statin | 1549686 | fluvastatin | Drug | RxNorm | NO | NO | NO |
|  | statin | 1551860 | pravastatin | Drug | RxNorm | NO | NO | NO |
|  | warfarin | 1310149 | warfarin | Drug | RxNorm | NO | NO | NO |

The SNOMED-CT for attrition of study population, eligibility criteria, comorbidities, echocardiographic findings, laboratory findings, and medication use are shown.

Abbreviations: SNOMED-CT, Systematized Nomenclature of Medicine Clinical Terms.

**Supplementary Table S2. Univariable linear regression analysis for the predictors of AS progression rate**

| **Variables** | **β** | **95% CI** | **P value** |
| --- | --- | --- | --- |
| Age (per +1 year) | 0.134 | 0.001 – 0.267 | 0.049 |
| Male sex | -0.383 | -3.552 – 2.787 | 0.813 |
| Hypertension | 2.271 | -1.260 – 5.802 | 0.207 |
| Diabetes mellitus | 4.284 | 0.121 – 8.448 | 0.044 |
| Dyslipidemia | 1.381 | -3.328 – 6.089 | 0.565 |
| Coronary artery disease | 2.881 | -1.093 – 6.856 | 0.155 |
| Heart failure | -10.009 | -15.488 – -4.529 | <0.001 |
| Atrial fibrillation | -6.028 | -11.032 – -1.023 | 0.018 |
| Stroke | -4.547 | -9.988 – 0.894 | 0.101 |
| Hemoglobin (per +1 g/dL) | 0.667 | -0.213 – 1.547 | 0.137 |
| Serum creatinine (per +1 mg/dL) | 0.975 | -0.061 – 2.011 | 0.065 |
| Glomerular filtration rate (per +1 mL/min/1.73m^2^) | -0.052 | -0.001 – 0.007 | 0.086 |
| Glomerular filtration rate <60 mL/min/1.73m^2^) | 2.464 | -1.281 – 6.210 | 0.197 |
| Fasting glucose (per +1 mg/dL) | 0.002 | -0.044 – 0.049 | 0.923 |
| Variability of fasting glucose during follow-up |  |  |  |
| - Standard deviation of fasting glucose (SD_FG_) | 0.000 | -0.105 – 0.105 | 0.999 |
| - Coefficient of variation of fasting glucose (CV_FG_) | -0.063 | -0.244 – 0.118 | 0.492 |
| - Average real variation of fasting glucose (ARV_FG_) | 0.101 | -0.020 – 0.222 | 0.100 |
| Total cholesterol (per +1 mg/dL) | 0.042 | -0.001 – 0.086 | 0.054 |
| Triglyceride (per +1 mg/dL) | 0.013 | -0.022 – 0.048 | 0.453 |
| HDL cholesterol (per +1 mg/dL) | 0.040 | -0.115 – 0.195 | 0.616 |
| LDL cholesterol (per +1 mg/dL) | -0.010 | -0.080 – 0.060 | 0.786 |
| HbA1c (per +1%) | 2.536 | 0.660 – 4.413 | 0.008 |
| HbA1c ≥7.0% | 3.802 | -1.929 – 9.534 | 0.193 |
| LVEDV (per +1 mL) | -0.063 | -0.105 – -0.020 | 0.004 |
| LVEF (per +1%) | 0.135 | -0.022 – 0.291 | 0.091 |
| LVEF <40% | -4.188 | -11.493 – 3.118 | 0.261 |
| LVMI (per +1 g/m2) | -0.032 | -0.078 – 0.013 | 0.162 |
| Vpeak (per +1 m/sec) | 4.847 | 2.142 – 7.552 | <0.001 |
| Vpeak ≥3.0 m/sec | 5.067 | 1.279 – 8.856 | 0.009 |
| meanPG (per +1 mmHg) | 0.494 | 0.305 – 0.682 | <0.001 |
| meanPG ≥30 mmHg | 7.668 | 1.638 – 13.699 | <0.001 |
| AVA (per +1 cm^2^) | -10.074 | -16.398 – -3.750 | 0.002 |
| AVA <1.5 cm^2^ | 7.318 | 1.424 – 13.212 | 0.015 |
| RAS blocker | -1.620 | -4.963 – 1.722 | 0.342 |
| Beta blocker | -2.955 | -6.801 – 0.890 | 0.132 |
| Calcium channel blocker | -0.183 | -3.898 – 3.531 | 0.923 |
| Spironolactone | -2.821 | -9.992 – 4.349 | 0.440 |
| Statins | 2.221 | -1.128 – 5.570 | 0.193 |
| Warfarin | -8.931 | -14.140 – -3.721 | 0.001 |
| DOAC | -8.885 | -18.615 – 0.845 | 0.073 |
| Antiplatelet agents | 3.813 | 0.449 – 7.176 | 0.026 |
| Insulin | 8.659 | -20.604 – 37.922 | 0.562 |
| Metformin | 0.773 | -4.878 – 6.424 | 0.788 |
| DPP4 inhibitors | -0.242 | -7.464 – 6.979 | 0.948 |
| SGLT2 inhibitors | 27.557 | -30.894 – 86.008 | 0.355 |
| Sulfonylurea | 1.125 | -5.458 – 7.708 | 0.737 |
| Thiazolidinedione | 6.032 | -17.880 – 29.943 | 0.621 |
| Alpha glucosidase inhibitor | 7.947 | -8.995 – 24.889 | 0.358 |

Because the value of the dependent variable was numerically small, the dependent variable (△Vpeak/year) was analyzed in units of ‘cm/sec/year’.

Univariable linear regression analyses were performed to investigate the association with the variables with the rate of AS progression (△Vpeak/year).

Abbreviations: HDL, high-density lipoprotein; LDL, low-density lipoprotein; LVEDV, left ventricular end-diastolic volume; LVEF, left ventricular ejection fraction; LVMI, left ventricular mass index; Vpeak, aortic valve maximal velocity; meanPG, mean pressure gradients across the aortic valve; AVA, aortic valve area; RAS, renin-angiotensin system; DOAC, direct oral anticoagulants; DPP4, dipeptidyl peptidase-4; SGLT2, sodium-glucose cotransporter 2.

**Supplementary Table S3. Univariable logistic regression analysis for the predictors of accelerated AS progression**

| **Variables** | **Unadjusted OR** | **95% CI** | **P value** |
| --- | --- | --- | --- |
| Age (per +1 year) | 1.010 | 0.999 – 1.020 | 0.068 |
| Male sex | 1.251 | 0.986 – 1.586 | 0.065 |
| Hypertension | 0.912 | 0.696 – 1.188 | 0.497 |
| Diabetes mellitus | 1.053 | 0.768 – 1.430 | 0.745 |
| Dyslipidemia | 0.845 | 0.582 – 1.206 | 0.364 |
| Coronary artery disease | 1.107 | 0.822 – 1.481 | 0.498 |
| Heart failure | 0.635 | 0.394 – 0.985 | 0.050 |
| Atrial fibrillation | 0.690 | 0.454 – 1.023 | 0.073 |
| Stroke | 0.717 | 0.455 – 1.095 | 0.136 |
| Hemoglobin (per +1 g/dL) | 1.047 | 0.983 – 1.117 | 0.154 |
| Serum creatinine (per +1 mg/dL) | 1.069 | 0.996 – 1.145 | 0.058 |
| Glomerular filtration rate (per +1 mL/min/1.73m^2^) | 0.998 | 0.994 – 1.002 | 0.298 |
| Glomerular filtration rate <60 mL/min/1.73m^2^) | 1.077 | 0.822 – 1.406 | 0.589 |
| Fasting glucose (per +1 mg/dL) | 0.997 | 0.994 – 1.001 | 0.124 |
| Variability of fasting glucose during follow-up |  |  |  |
| - Standard deviation of fasting glucose (SD_FG_) | 1.000 | 0.991 – 1.010 | 0.986 |
| - Coefficient of variation of fasting glucose (CV_FG_) | 0.995 | 0.979 – 1.011 | 0.539 |
| - Average real variation of fasting glucose (ARV_FG_) | 1.002 | 0.992 – 1.013 | 0.645 |
| Total cholesterol (per +1 mg/dL) | 1.003 | 1.000 – 1.006 | 0.051 |
| Triglyceride (per +1 mg/dL) | 1.003 | 1.000 – 1.005 | 0.034 |
| HDL cholesterol (per +1 mg/dL) | 1.001 | 0.990 – 1.013 | 0.823 |
| LDL cholesterol (per +1 mg/dL) | 1.003 | 0.998 – 1.009 | 0.197 |
| HbA1c (per +1%) | 1.175 | 1.036 – 1.332 | 0.012 |
| HbA1c ≥7.0% | 1.312 | 0.887 – 1.925 | 0.168 |
| LVEDV (per +1 mL) | 0.998 | 0.995 – 1.002 | 0.333 |
| LVEF (per +1%) | 0.999 | 0.987 – 1.011 | 0.820 |
| LVEF <40% | 1.048 | 0.594 – 1.779 | 0.865 |
| LVMI (per +1 g/m2) | 1.001 | 0.997 – 1.004 | 0.667 |
| Vpeak (per +1 m/sec) | 1.673 | 1.376 – 2.034 | <0.001 |
| Vpeak ≥3.0 m/sec | 1.867 | 1.423 – 2.444 | <0.001 |
| meanPG (per +1 mmHg) | 1.039 | 1.026 – 1.053 | <0.001 |
| meanPG ≥30 mmHg | 1.725 | 1.134 – 2.598 | 0.010 |
| AVA (per +1 cm^2^) | 0.366 | 0.218 – 0.593 | <0.001 |
| AVA <1.5 cm^2^ | 1.865 | 1.254 – 2.805 | 0.002 |
| RAS blocker | 0.727 | 0.560 – 0.938 | 0.015 |
| Beta blocker | 0.810 | 0.599 – 1.085 | 0.164 |
| Calcium channel blocker | 0.847 | 0.635 – 1.122 | 0.252 |
| Spironolactone | 0.525 | 0.266 – 0.954 | 0.046 |
| Statins | 1.069 | 0.831 – 1.371 | 0.602 |
| Warfarin | 0.513 | 0.318 – 0.797 | 0.004 |
| DOAC | 0.601 | 0.241 – 1.304 | 0.230 |
| Antiplatelet agents | 1.031 | 0.800 – 1.325 | 0.812 |
| Insulin | 7.880 | 1.005 – 159.575 | 0.074 |
| Metformin | 0.806 | 0.510 – 1.239 | 0.340 |
| DPP4 inhibitors | 0.846 | 0.469 – 1.452 | 0.558 |
| SGLT2 inhibitors | N/A | N/A | N/A |
| Sulfonylurea | 1.046 | 0.631 – 1.684 | 0.856 |
| Thiazolidinedione | 1.306 | 0.180 – 6.719 | 0.759 |
| Alpha glucosidase inhibitor | 0.519 | 0.080 – 1.981 | 0.399 |

Univariable logistic regression analyses were performed to investigate the association with the variables with the accelerated AS progression, defined as the △Vpeak/year of >0.2 m/sec/year.

Abbreviations: HDL, high-density lipoprotein; LDL, low-density lipoprotein; LVEDV, left ventricular end-diastolic volume; LVEF, left ventricular ejection fraction; LVMI, left ventricular mass index; Vpeak, aortic valve maximal velocity; meanPG, mean pressure gradients across the aortic valve; AVA, aortic valve area; RAS, renin-angiotensin system; DOAC, direct oral anticoagulants; DPP4, dipeptidyl peptidase-4; SGLT2, sodium-glucose cotransporter 2; N/A, not applicable.
